# Swainsonine protects both murine and human haematopoietic systems from chemotherapeutic toxicity

**DOI:** 10.1038/sj.bjc.6690326

**Published:** 1999-04

**Authors:** J-L D Klein, J D Roberts, M D George, J Kurtzberg, P Breton, J-C Chermann, K Olden

**Affiliations:** Laboratory of Molecular Carcinogenesis, National Institute of Environmental Health Sciences, National Institutes of Health, Research Triangle Park, NC 27709, USA; Pediatric Bone Marrow Laboratory, Duke University Hospital, Durham, NC 27710, USA; Laboratoires UPSA, BP 325, 92506 Rueil-Malmaison, Cedex, France; INSERM U322, BP 33, F 13273 Marseille, Cedex 9, France

**Keywords:** swainsonine, cancer, bone marrow, cyclophosphamide, AZT

## Abstract

The haematopoietic system is sensitive to cytotoxic damage and is often the site of dose-limiting toxicity. We previously reported that swainsonine, an inhibitor of protein glycosylation, reduced the bone marrow toxicity resulting from a single dose of anticancer drugs in otherwise healthy mice. However, more important questions are (1) can swainsonine protect tumour-bearing mice without interfering with the anti-tumour effects of the drugs, and (2) can swainsonine stimulate haematopoietic activity of human, as well as murine, bone marrow. We demonstrate here that swainsonine protects C57BL/6 mice bearing melanoma-derived tumours from cyclophosphamide-induced toxicity without interfering with the drug's ability to inhibit tumour growth. Similar results were obtained in vivo with 3́-azido-3́-deoxythymidine (AZT), a myelosuppressive agent often used in therapy for acquired immune deficiency syndrome. Swainsonine increased both total bone marrow cellularity and the number of circulating white blood cells in mice treated with doses of AZT that typically lead to severe myelosuppression. Swainsonine also increased the number of erythroid and myeloid colony forming cells (CFCs) in short-term cultures of murine bone marrow, restoring the number of progenitor cells to the control level in the presence of AZT doses that reduced CFCs by 80%. With respect to the sensitivity of human haematopoietic cells to swainsonine, we show that swainsonine protected human myeloid progenitor cells from AZT toxicity in vitro. These results suggest that swainsonine may be useful as an adjuvant in several types of human chemotherapy. © 1999 Cancer Research Campaign


British Journal of Cancer (1999) 80(1/2), 87–95
© 1999 Cancer Research Campaign
Article no. bjoc.1998.0326

### Swainsonine protects both murine and human haematopoietic systems from chemotherapeutic toxicity

**J-LD Klein[1,*], JD Roberts[1], MD George[1], J Kurtzberg[2], P Breton[3], J-C Chermann[4] and K Olden[1]**

[1]Laboratory of Molecular Carcinogenesis, National Institute of Environmental Health Sciences, National Institutes of Health, Research Triangle Park, NC 27709, USA; [2]Pediatric Bone Marrow Laboratory, Duke University Hospital, Durham, NC 27710, USA; [3]Laboratoires UPSA, BP 325, 92506 Rueil-Malmaison, Cedex, France; [4]INSERM U322, BP 33, F 13273 Marseille, Cedex 9, France

**Summary** The haematopoietic system is sensitive to cytotoxic damage and is often the site of dose-limiting toxicity. We previously reported that swainsonine, an inhibitor of protein glycosylation, reduced the bone marrow toxicity resulting from a single dose of anticancer drugs in otherwise healthy mice. However, more important questions are (1) can swainsonine protect tumour-bearing mice without interfering with the anti-tumour effects of the drugs, and (2) can swainsonine stimulate haematopoietic activity of human, as well as murine, bone marrow. We demonstrate here that swainsonine protects C57BL/6 mice bearing melanoma-derived tumours from cyclophosphamide-induced toxicity without interfering with the drug's ability to inhibit tumour growth. Similar results were obtained in vivo with 3´-azido-3´-deoxythymidine (AZT), a myelosuppressive agent often used in therapy for acquired immune deficiency syndrome. Swainsonine increased both total bone marrow cellularity and the number of circulating white blood cells in mice treated with doses of AZT that typically lead to severe myelosuppression. Swainsonine also increased the number of erythroid and myeloid colony forming cells (CFCs) in short-term cultures of murine bone marrow, restoring the number of progenitor cells to the control level in the presence of AZT doses that reduced CFCs by 80%. With respect to the sensitivity of human haematopoietic cells to swainsonine, we show that swainsonine protected human myeloid progenitor cells from AZT toxicity in vitro. These results suggest that swainsonine may be useful as an adjuvant in several types of human chemotherapy.

**Keywords:** swainsonine; cancer; bone marrow; cyclophosphamide; AZT

A primary strategy of cancer chemotherapy is to administer cytotoxic drugs to destroy malignant tumour cells at doses that do not compromise the function of healthy tissues critical for the survival of the patient. Unfortunately, the structural or behavioural differences between normal and malignant cells are not absolute so that the selectivity of cytotoxic agents is relative. Epithelial linings, such as the oropharynx and the gastrointestinal tract, and the bone marrow are among the most sensitive tissues (Dorr and Fritz, 1980; Hoagland, 1992; Perry, 1992; Pratt et al, 1994). In fact, myelosuppression is often the dose-limiting feature in chemotherapy regimens for a number of diseases, including cancer (Hoagland, 1992) and acquired immune deficiency syndrome (AIDS) (Richman et al, 1987; Shaunak and Bartlett, 1989; Walker et al, 1987; McLeod and Hammer, 1992). Supporting patients through a period of myelosuppression or decreased resistance to infection has become a significant part of a chemotherapeutic regimen (Vose and Armitage, 1992).

Several strategies have been deployed in recent years to overcome the side-effects of chemotherapy with much of the attention focused on stimulation of the haematopoietic system. Haematopoietic cytokines, such as interleukin-2, granulocyte colony-stimulating factor (G-CSF), granulocyte–macrophage colony-stimulating factor (GM-CSF), and erythropoietin (Epo) have been used to stimulate recovery of bone marrow function in patients receiving chemotherapy (Fischl et al, 1990; Davey et al, 1991; Laporte et al, 1991; Aviles et al, 1995) with G-CSF yielding the most clinical utility so far (for example, see Donadieu et al, 1997 and Gisselbrecht et al, 1997). Unfortunately, these haematopoietic growth factors have significant side-effects at standard doses and provide only limited protection. For example, approximately 30% of AIDS patients who receive 3′-azido-3′-deoxythymidine (AZT) in combination with haematopoietic growth factors still require blood transfusions partly due to the drug-induced myelosuppression (Miles, 1992). Recent studies with negative haematopoietic regulatory factors, such as macrophage inflammatory protein-1α (Lord et al, 1992; Hunter et al, 1995) and the tetrapeptide acetyl-*N-Ser-Asp-Lys-Pro* (Aidoudi et al, 1996; Watanabe et al, 1996), have shown promise as important haemoprotective agents during chemotherapy. However, additional clinical trials with these agents are needed to confirm their utility.

Earlier studies in our laboratory suggested that swainsonine, a protein glycosylation inhibitor known to block metastasis in experimental systems, may be effective in stimulating bone marrow recovery with less severe side-effects than G-CSF or GM-CSF (Oredipe et al, 1991). However, two important issues remain to be addressed. First, do the protective effects of swainsonine administration limit the effectiveness of cytoreductive chemotherapeutic drugs? Second, although studies in mouse model systems are important in elaborating the functional potential of swainsonine as a hematopoietic growth factor, the relevance of the data to

*Received 7 October 1997*
*Revised 13 August 1998*
*Accepted 14 August 1998*

*Correspondence to:* JD Roberts

**Current Address:* PO Box 13398, BioMet Discovery, Glaxo Wellcome, Inc., Research Triangle Park, NC 27709, USA

clinical application has not yet been established. If the mouse data are confirmed with human bone marrow, swainsonine may offer promise in future intensive chemotherapy programmes, allowing increased dosage and/or frequency of administration of cytotoxic agents without increasing toxic effects to the haematopoietic system.

We report here that swainsonine does not reduce the effectiveness of cytoreductive chemotherapeutic drugs and that swainsonine has protective effects on certain classes of both murine and human blood cells.

### MATERIALS AND METHODS

### Materials

Preparations of swainsonine used in these studies were chemically synthesized by and purchased from Toronto Research Chemicals, Inc. (North York, Ontario, Canada). Interleukin-3 and Epo were obtained from R&D Systems (Minneapolis, MN, USA) and reconstituted according to the manufacturer's directions. AZT was synthesized by Raylo Chemical Co. (Edmonton, Alberta, Canada) and obtained through the Chemistry Support Group at NIEHS. Cyclophosphamide, purchased as Cytoxan® for injection (Bristol Myers, Princeton, NJ, USA), was freshly prepared before use by dissolving the powder in sterile non-pyrogenic 0.9% sodium chloride solution to yield a 1 mg $ml^{-1}$ solution, as recommended by the supplier.

### Animals

Pathogen-free female C57BL/6 mice, 7–9 weeks old, were obtained from Charles River Laboratories, Raleigh, NC, USA, and similar female B6C3F1 mice were obtained from the National Cancer Institute Animal Program, Bethesda, MD, USA. Mice were allowed at least 1 week to acclimate prior to experimentation and were fed a standard diet in a humidity- and temperature-controlled room. The animals were handled according to institutional ethical guidelines.

### B16-F1 cell culture

B16-F1 murine melanoma cells (Dr IJ Fidler, The University of Texas MD Anderson Cancer Center, Houston, TX, USA) were grown in Dulbecco's modified Eagle's medium (DMEM; Gibco BRL, Life Technologies, Grand Island, NY, USA) supplemented with 5% Fetal Clone II (HyClone Laboratories, Inc., Logan, UT, USA), sodium pyruvate (1 mM), non-essential amino acids (0.1 mM), MEM vitamin solution (1 X), L-glutamine (2 mM), penicillin (50 IU $ml^{-1}$) and streptomycin (50 μg $ml^{-1}$). Cells were grown as monolayers in tissue culture flasks in a humidified atmosphere of 5% $CO_2$–95% air at 37°C. Cells to be used for inoculation were removed from flasks by treatment with 0.25% trypsin in 0.02% EDTA and were washed twice with $Ca^{2+}Mg^{2+}$-free phosphate-buffered saline solution (PBS) before injection into mice.

### Cell plating efficiency

Cells were plated at 200 cells per each 60 mm dish in DMEM supplemented with 5% fetal bovine serum, L-glutamine (2 mM), penicillin (100 IU $ml^{-1}$) and streptomycin (100 μg $ml^{-1}$). Cyclophosphamide (0, 0.1, 0.5, 0.75, 1 or 2 mM) and swainsonine (0, 3, 10 or 50 μg $ml^{-1}$) were added to the medium just prior to addition of the cells. Cells were grown as described above for 8 days, washed once with PBS, fixed 15 min in methanol, air dried, stained with 4% Giemsa stain, air dried and scored. A cluster of 15 or more cells was counted as a colony. Five plates were used for each point.

### Generation of experimental tumours in C57BL/6 mice

Mice were inoculated subcutaneously in the upper back with $5 \times 10^4$ viable B16-F1 melanoma cells in 0.2 ml of sterile non-pyrogenic 0.9% sodium chloride solution. Animals developed palpable tumours within 12–14 days. Mice were injected intraperitoneally (i.p.) with swainsonine (3 μg) or saline twice daily for 10 consecutive days beginning 1 day prior to the administration of cyclophosphamide, that is, day –1 when a single dose of cyclophosphamide was given and days –1 and 13 when two doses of cyclophosphamide were given. Cyclophosphamide-treated mice received a single i.p. injection of cyclophosphamide (500 mg $kg^{-1}$ body weight) on day 0 only, or on day 0 and day 14. Animals bearing tumours were euthanized when appropriate according to NIEHS guidelines. Tumours were measured every 2–3 days with a caliper (Biomedical Research Instruments, Rockville, MD, USA). Tumour volumes were calculated using the formula: $V = 4/3\,\pi\,(r_1^2 \times r_2)$ where $r_1$ and $r_2$ are the minor and major radii respectively.

### Murine bone marrow cellularity

Bone marrow cell suspensions were prepared under sterile conditions using both femora and tibiae from each mouse. Tissue was removed aseptically from bones, the ends were opened, and the bone marrow cells were flushed from the marrow cavities with a sterile syringe and a 26-gauge needle with 10 ml of ice-cold RPMI-1640 medium (Gibco BRL) supplemented with 3% bovine serum albumin (BSA; Sigma, St Louis, MO, USA). Cells were then collected by centrifugation and resuspended in 10 ml of ice-cold RPMI. Single cell suspensions were obtained by initially passing cell suspensions through a 22-gauge needle, then again through a 25-gauge needle. The remaining cell clusters were eliminated by filtering the cell suspensions through a 70 μm nylon mesh (Falcon, Franklin Lakes, NJ, USA). Cells were counted with a Coulter Counter ZM (Coulter Corp., Miami, FL, USA). Numbers are reported as total nucleated bone marrow cells per two posterior legs (two femora and two tibiae).

### Mouse bone marrow progenitor cell assay

Bone marrow progenitor cells were grown using either the Mouse Bone Marrow Stem Cell Differentiation kit from Gibco BRL or the MethoCult™ M3330 or M3230 media from StemCell Technologies, Inc. (Vancouver, BC, Canada). For the Mouse Bone Marrow Stem Cell Differentiation kit, Epo (2 U) and interleukin-3 (75 U) were added to the 0.75 ml progenitor cell growth medium containing methylcellulose. Mononuclear bone marrow cells (0.25 ml at $0.5 \times 10^6$ cells per ml) were then added, mixed by vortexing, and aliquoted (0.5 ml per well) into a 24-well tissue culture dish. The cells were incubated for 14 days in a humidified incubator at 37°C and 5% carbon dioxide.

The MethoCult™ M3330 medium contains fetal bovine serum (30%) and Epo (10 ng $ml^{-1}$). This medium is designed for the

© Cancer Research Campaign 1999

growth of early erythroid progenitor cells (E-CFC) from murine bone marrow. The MethoCult™ M3230 contains 30% fetal bovine serum and does not contain any added growth factors; it is designed for the growth of murine granulocyte–macrophage progenitor cells (GM-CFC). For either medium, 0.1 ml of bone marrow cells (at $2 \times 10^6$ or $5.6 \times 10^5$ cells per ml for M3330 or M3230 respectively) was added to 0.9 ml of the prepared methylcellulose medium. The cells and medium were then mixed with a vortex, transferred to a 35-mm dish and placed in a humidified incubator at 37°C and 5% carbon dioxide. Early E-CFC were scored after 2 days of incubation; GM-CFC were scored after 7 days. According to the manufacturer's instructions, 200–300 early E-CFC should be present when $2 \times 10^5$ nucleated bone marrow cells are plated in M3330 medium. With the M3230 medium, 5–20 GM-CFC colonies are typically scored when $5.6 \times 10^4$ nucleated bone marrow cells are plated.

### AZT and swainsonine treatment

Groups of 10 B6C3F1 female mice received AZT dissolved in methylcellulose, administered by oral gavage twice daily (400 mg AZT per kg body weight per day, total dose). Swainsonine, dissolved in sterile saline, was administered by i.p. injection twice daily (1 mg $kg^{-1}$ body weight total daily dose, 0.2 ml per injection) beginning on the same day as AZT treatment and continuing until the animal was sacrificed. Blood samples were obtained from the retro-orbital sinuses of animals anaesthetized with isoflurane and dioxygen. Complete blood counts and white blood cell differential counts were generated using the Technicon H*1 haematology analyser (Miles, Inc., Tarrytown, NY, USA). Wright–Giemsa stained smears and spun microhaematocrits were prepared for each sample. Reticulocyte counts were obtained from blood smears stained with new methylene blue. White blood cell differential counts were performed with a light microscope on at least two samples from each group for comparison with the automated counts and for evaluating cell morphology. All H*1 differential counts that were determined to be abnormal were confirmed by manual counts using the prepared smears.

### Human bone marrow progenitor cell assay

Human bone marrow samples were obtained either on the occasion of a bone marrow allograft by aspiration from the iliac crest of a healthy donor or from a patient in complete remission after a haematological disease. Cells ($5 \times 10^4$ mononuclear human bone marrow cells) were plated in complete methylcellulose medium (MethoCult™ H4433, StemCell Technologies, Inc.) in the presence or absence of AZT or swainsonine. Colonies were identified and scored with an inverted microscope after 14 days incubation at 37°C and 5% carbon dioxide in a humidified atmosphere as described (Calenda et al, 1992*a*, 1992*b*, 1993). Each sample was plated in triplicate.

The isobologram analysis, described by Elion et al (1954), was used to test for the antagonistic or synergistic behaviour of compounds administered simultaneously. In the experiments with human bone marrow progenitor cells, we tested various concentrations of AZT (0.1, 1, 10 and 100 μM) in combination with various concentrations of swainsonine (1, 10, 50 and 100 μg $ml^{-1}$). This experimental design generates a series of growth curves for each concentration of AZT associated with a concentration of swainsonine. Fractional inhibitory concentrations (FIC) for AZT and swainsonine were then calculated by dividing the concentration of the first compound present in the combination by the amount of the second drug, which is required to give 95% inhibition of cell growth. The FICs of pairs of drugs were then plotted and compared to the line connecting unity on the ordinate to unity on the abscissa. This line represents a strictly additive effect of the two compounds. Points above and to the right of this line indicate antagonism between two drugs, whereas points below and to the left of this line represent synergism.

### Leukaemia and lymphoma cell lines

The human leukaemia cell lines HL-60 and Jurkat were obtained from the ATCC (Rockville, MD, USA) and were grown in RPMI-1640 medium (Gibco BRL) supplemented with 20% Fetal Clone II, L-glutamine (2 mM), penicillin (100 IU $ml^{-1}$) and streptomycin (100 μg $ml^{-1}$). WEHI 3B, a mouse leukaemia cell line was the kind gift of Dr A R Migliaccio (New York Blood Center, New York, NY, USA) and was cultured in RPMI medium, supplemented with 10% Fetal Clone II, L-glutamine (2 mM), penicillin (100 IU $ml^{-1}$) and streptomycin (100 μg $ml^{-1}$). WR19L, a mouse lymphoma cell line was obtained from ATCC and was grown in DMEM supplemented with 10% Fetal Clone II, L-glutamine (2 mM), penicillin (100 IU $ml^{-1}$) and streptomycin (100 μg $ml^{-1}$). All cell lines were tested and found to be negative for mycoplasma.

For the methylcellulose assay, cells were plated either in 'complete' methylcellulose medium MethoCult™ M3434 (StemCell Technologies, Inc.) which contains 0.9% methylcellulose in Iscove's MDM, 15% fetal bovine serum, BSA (1%), bovine pancreatic insulin (10 μg $ml^{-1}$), human transferrin (200 μg $ml^{-1}$), 2-mercaptoethanol (0.1 mM), L-glutamine (2 mM), recombinant murine interleukin-3 (10 ng $ml^{-1}$), recombinant human interleukin-6 (10 ng $ml^{-1}$), recombinant murine stem cell factor (50 ng $ml^{-1}$) and recombinant human Epo (3 units per ml), or in MethoCult™ M3230 (also from StemCell Technologies, Inc.) which contains 0.9% methylcellulose in α-MEM, fetal bovine serum (30%), BSA (1%), 2-mercaptoethanol (0.1 mM) and glutamine (2 mM). Colonies consisting of more than 30 cells were scored 7–14 days later, depending on the cell line.

### Statistics

Tumour sizes in mice were compared using an analysis of variance on the log-transformed volumes of the subcutaneous mass. In other experiments, *P*-values were calculated using either Student's two-tailed paired *t*-test or Student's one-tailed paired *t*-test, as appropriate.

### RESULTS

### Tumour cell killing capacity of cyclophosphamide in presence of swainsonine

B16-F1 cells were chosen as the tumour model to see whether swainsonine influenced the cytotoxicity of cyclophosphamide either in vitro or in vivo. To examine the effects of swainsonine on cyclophosphamide toxicity in vitro, the plating efficiency of B16-F1 cells was examined by co-exposing cells to cyclophosphamide and swainsonine, to cyclophosphamide plus the swainsonine buffer, or to swainsonine plus the cyclophosphamide buffer. The dose of cyclophosphamide used in this experiment (750 μM)

© Cancer Research Campaign 1999

**Table 1** Effect of swainsonine and cyclophosphamide on the plating efficiency of B16-F1 murine melanoma cells

| Swainsonine | Plating efficiency (%) Cyclophosphamide | |
|---|---|---|
| | 0 μM | 750 μM |
| 0 μg ml$^{-1}$ | 32 ± 4 | 18 ± 2 |
| 3 μg ml$^{-1}$ | 31 ± 4 | 13 ± 1 |
| 10 μg ml$^{-1}$ | 29 ± 5 | 15 ± 3 |
| 50 μg ml$^{-1}$ | 32 ± 2 | 14 ± 3 |

B16-F1 murine melanoma cells were plated at 200 cells per dish as described in Materials and Methods. After 8 days, colonies of 15 cells or more were scored. Five plates were used for each variable. Numbers are the means ± standard deviation.

was determined from a similar experiment in which concentrations up to 2.5 mM were tested; 750 μM cyclophosphamide-treated B16-F1 melanoma cells gave rise to a plating efficiency approximately one-half that of the control (Table 1). The addition of swainsonine had no effect on the plating efficiency of the cells in the absence of cyclophosphamide at any of the concentrations tested (Table 1). This is consistent with previous findings that swainsonine does not interfere with cell growth in vitro (Humphries et al, 1986). In the presence of a cyclophosphamide dose that significantly reduced plating efficiency of the tumour cells, swainsonine appeared to cause a very slight decrease in the number of colonies (Table 1). These results indicate that swainsonine does not interfere with the cytotoxicity of cyclophosphamide in vitro.

To determine whether swainsonine alters the toxicity of cyclophosphamide in vivo, mice bearing palpable tumours (ten mice per group) were exposed to both agents using a clinically-relevant regimen. On day zero, animals were given a single i.p. injection of cyclophosphamide (500 mg per kg body weight) or solvent buffer (saline). The i.p. administration of swainsonine (3 μg in 0.2 ml saline) was initiated 1 day prior (day –1) to injection of cyclophosphamide and was continued at a frequency of two injections per day for 10 consecutive days. Tumours grew rapidly in animals that received either saline or swainsonine alone (Figure 1) and tumours in the two groups were similar in size. However, tumours in mice that received cyclophosphamide (Figure 1) remained small and relatively constant in size for about 2 weeks after injection of cyclophosphamide. Tumours in mice that received both cyclophosphamide and swainsonine were slightly larger than those in the cyclophosphamide-treated mice (see Figure 1A, day 9), primarily due to the required euthanasia on day 9 of several mice in the cyclophosphamide group which had the largest tumours (the euthanasia was necessitated by NIEHS criteria for morbidity, including weight loss, tumour ulceration and prolonged unhealthy appearance). An analysis of variance of the log-transformed tumour volumes indicated that the inhibition of tumour size by cyclophosphamide was highly significant ($P < 0.001$), that swainsonine alone had no effect on tumour size and that, in the presence of swainsonine, cyclophosphamide continued to have highly significant inhibitory effect on tumour growth ($P < 0.001$).

Because there was an increase in tumour size in both the cyclophosphamide and cyclophosphamide plus swainsonine groups after day 14, another experiment was performed in which a second dose of cyclophosphamide, with or without swainsonine,

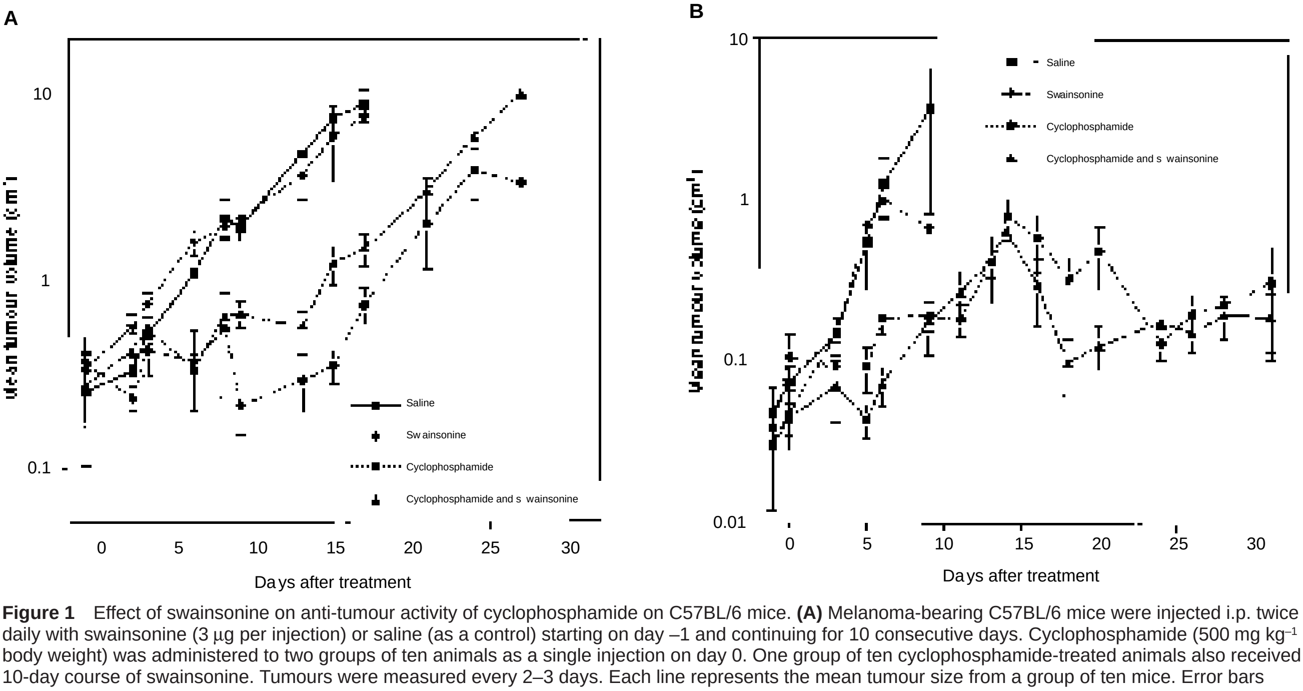

**Figure 1** Effect of swainsonine on anti-tumour activity of cyclophosphamide on C57BL/6 mice. **(A)** Melanoma-bearing C57BL/6 mice were injected i.p. twice daily with swainsonine (3 μg per injection) or saline (as a control) starting on day –1 and continuing for 10 consecutive days. Cyclophosphamide (500 mg kg$^{-1}$ body weight) was administered to two groups of ten animals as a single injection on day 0. One group of ten cyclophosphamide-treated animals also received a 10-day course of swainsonine. Tumours were measured every 2–3 days. Each line represents the mean tumour size from a group of ten mice. Error bars represent the standard errors. Statistical analysis indicated that there was no difference in tumour sizes between saline- and swainsonine-treated animals, a highly significant decrease in tumour size in the cyclophosphamide-treated group ($P < 0.001$), a highly significant decrease in the tumour size of cyclophosphamide with swainsonine-treated mice ($P < 0.001$) and a slight difference between cyclophosphamide and cyclophosphamide with swainsonine-treated groups ($P < 0.05$). **(B)** Melanoma-bearing C57BL/6 mice were treated as in **A**, except that a second dose of cyclophosphamide (500 mg kg$^{-1}$ body weight) was given at day 14 to the surviving mice from the two groups that initially received cyclophosphamide at day 0. Animals that initially received both cyclophosphamide and swainsonine were also given an additional course of swainsonine (10 days, twice daily i.p. injections of 3 μg swainsonine per injection) starting at day 13. Error bars represent the standard errors. In this experiment, there was no difference between saline- and swainsonine-treated animals, a significant decrease in tumour size in both the cyclophosphamide- and cyclophosphamide with swainsonine-treated animals ($P < 0.01$), and no difference between the cyclophosphamide- and cyclophosphamide with swainsonine-treated groups ($P > 0.05$)

© Cancer Research Campaign 1999

**Table 2** Effect of swainsonine on haematological parameters in AZT-treated mice

| Treatment | WBC (cells μl$^{-1}$) | Lymphocytes (cells μl$^{-1}$) | Monocytes (cells μl$^{-1}$) | RBC (mil μl$^{-1}$) | HGB (g dl$^{-1}$) | HCT (%) | MCV (fl) | MCH (pg) | MCHC (g dl$^{-1}$) | Platelets (1000 μl$^{-1}$) | Reticulocytes (1000 μl$^{-1}$) |
|---|---|---|---|---|---|---|---|---|---|---|---|
| Vehicles | 4250± 340 | 3090± 310 | 130± 20 | 8.1± 0.1 | 13.1± 0.3 | 42.3± 0.5 | 52.0± 0.6 | 16.2± 0.4 | 31.1± 0.5 | 1080± 80 | 150± 30 |
| AZT | [a]2790 ± 180 | [a]2300 ± 150 | [a]33 ± 6 | [a]5.4 ± 0.6 | [a]10 ± 1 | [a]31 ± 4 | [a]57± 1 | [a]18.4 ± 0.3 | [a]32.2 ± 0.4 | [a]1680 ± 80 | [a]49 ± 27 |
| Sw | 4110 ± 460 | 3130 ± 380 | 110± 10 | [a]8.8 ± 0.1 | [a]14.6 ± 0.1 | [a]46.2 ± 0.4 | 52.4 ± 0.4 | 16.6 ± 0.1 | 31.7 ± 0.3 | 1120 ± 80 | [a]100 ± 20 |
| AZT/Sw | [b]4360 ± 580 | [b]3820 ± 540 | [a]55 ± 10 | [a]5.4 ± 0.7 | [a]10 ± 1.5 | [a]32 ± 5 | [a]57 ± 2 | [a]18.4 ± 0.5 | 32.0 ± 0.5 | [a]1900 ± 100 | [a]30 ± 13 |

Animals were treated with AZT (400 mg kg$^{-1}$ body weight) twice daily by oral gavage and with swainsonine (Sw, 1 mg kg$^{-1}$ body weight) twice daily by i.p injection as described in the Materials and Methods. Animals were bled on day 23 and cells were counted and identified by the Technicon H*1 haematology analyser and Wright–Giemsa stained smears. Each number represents the means ± standard error of 6–10 animals. Abbreviations: WBC, total leucocytes; RBC, erythrocytes; HGB, haemoglobin; HCT, haematocrit; MCV, mean corpuscular volume; MCH, mean corpuscular haemoglobin; MCHC, mean corpuscular haemoglobin concentration. [a]Significantly different from vehicle control value, $P < 0.05$. [b]Significantly different from AZT-treated value, $P < 0.05$.

was given at day 14 to the surviving mice (Figure 1B). As before, the cyclophosphamide-treated animals had much smaller tumours than mice treated with saline or swainsonine alone during the first course of treatment ($P < 0.01$). (None of the saline- or swainsonine-treated animals survived to day 14, and thus, were not candidates for the second dose.) The data showed that a second round of chemotherapy extended the time during which the growth of the B16-F1-derived tumours was inhibited (Figure 1B). Furthermore, administration of swainsonine for 10 days during the second round of cyclophosphamide treatment had no effect on the reduction in growth of the tumours from days 14 to 31. These results indicated that swainsonine did not compromise the therapeutic index of cyclophosphamide, allowing the anti-tumour drug to slow tumour growth through at least two cycles of therapy. Taken together with the in vitro results, this demonstrates that swainsonine does not interfere with the antineoplastic activity of cyclophosphamide toward B16-F1 melanoma tumour cells.

During the course of these experiments, we noted the days following treatment on which animals had to be euthanized due to NIEHS guidelines regarding either tumour size, overall weight loss, or general morbidity. All tumour-bearing animals given saline alone or swainsonine alone had to be euthanized within 17 days of the initiation of treatment in the first experiment (Figure 1A) and within 10 days in the second experiment (Figure 1B). On the other hand, only 33% of tumour-bearing animals that received cyclophosphamide had to be euthanized during this same period, and this number was reduced to 20% when swainsonine was co-administered with cyclophosphamide. This observation is consistent with previously published studies (Oredipe et al, 1991) that showed swainsonine reduced the toxicity of cyclophosphamide in non-tumour-bearing mice.

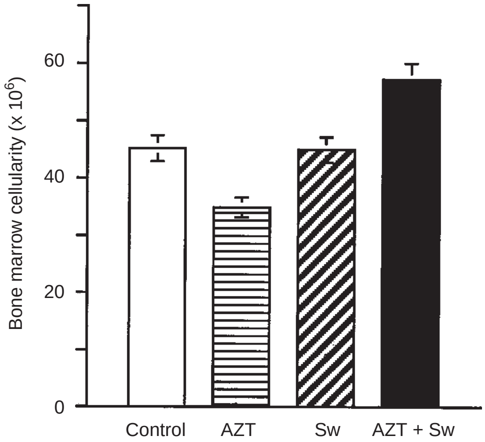

**Figure 2** Effect of swainsonine on bone marrow cellularity of AZT-treated B6C3F1 mice. AZT (400 mg kg$^{-1}$ body weight per day) was dissolved in methylcellulose and given by oral gavage in two equal doses per day. Swainsonine (0.2 ml per injection, 1 mg kg$^{-1}$ body weight per day) was injected i.p. twice a day with the first dose administered immediately following the administration of the AZT. Twenty-three days after the beginning of the treatment, three animals per group were sacrificed and the bone marrow cells from both femora and tibiae were flushed out and counted. Numbers given are the mean nucleated cells per two femora and two tibiae. The difference between AZT-treated mice and the control group was statistically significant ($P < 0.05$ using one-tailed *t*-test, used because previous data have shown that AZT treatment reduces bone marrow cellularity); the increase in cellularity due to swainsonine was also statistically significant ($P < 0.01$ for AZT alone vs AZT plus swainsonine using a two-tailed *t*-test).

### Effect of swainsonine on the haematopoietic toxicity of AZT

Prior studies have examined the effects of swainsonine on four chemical agents used in the treatment of cancer; therefore, we considered whether swainsonine is effective in protecting the bone marrow against myelosuppressive agents used in the treatment of AIDS. We focused on AZT because it is one of the most commonly used drugs in the treatment of AIDS and because its limiting toxicity is haematopoietic (Yarchoan et al, 1989; McLeod and Hammer, 1992).

We used the mouse model in these studies because it has been reported that female B6C3F1 mice that received AZT twice a day by oral gavage developed myelosuppression (Thompson et al, 1991). In our study, AZT was administered by gavage for 23 consecutive days; control animals received methylcellulose without AZT and i.p. injection of saline instead of swainsonine. AZT treatment resulted in a significant decrease in bone marrow cellularity after 15 days ($39 \pm 4 \times 10^6$ cells in two posterior legs vs $57 \pm 5 \times 10^6$ cells in two posterior legs for treated and control animals respectively). Similar results were obtained after 23 days of treatment (Figure 2). Treatment of healthy animals with swainsonine had no apparent effect on bone marrow cellularity. However, co-treatment with AZT and swainsonine led to a significant increase in bone marrow cellularity.

AZT-induced myelosuppression was also evident in peripheral blood cell counts. Animals treated with AZT for as little as 5 days

© Cancer Research Campaign 1999

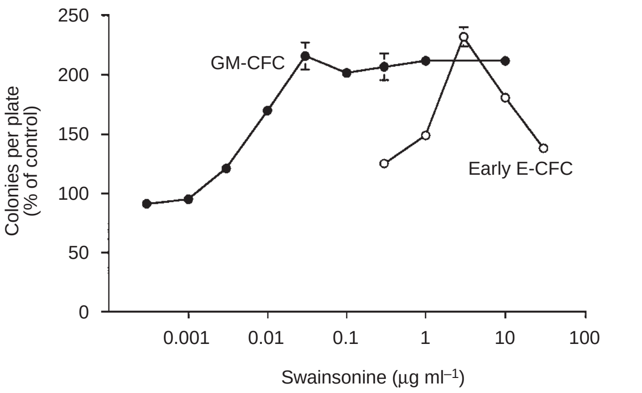

**Figure 3** Effect of swainsonine on murine bone marrow colony-forming units. Bone marrow cells from C57BL/6 mice were isolated and cultured in methylcellulose as described. Swainsonine was added to the methylcellulose medium at the concentrations indicated on the graph. For early E-CFC, $3 \times 10^5$ nucleated bone marrow cells were plated in M3330 medium and incubated for 2 days, at which time erythroid colonies were identified and scored. Data are expressed as a percentage of the control and are the average of three experiments (except for 0.3 and 30 µg ml$^{-1}$ of swainsonine, which are each the average of two experiments). For GM-CFC, $5.6 \times 10^4$ bone marrow cells were plated in M3230 medium and incubated for 7 days, at which time GM-CFC colonies were identified and scored. Each value is the average of three plates and is expressed as a percentage of the control. Bars represent the standard error

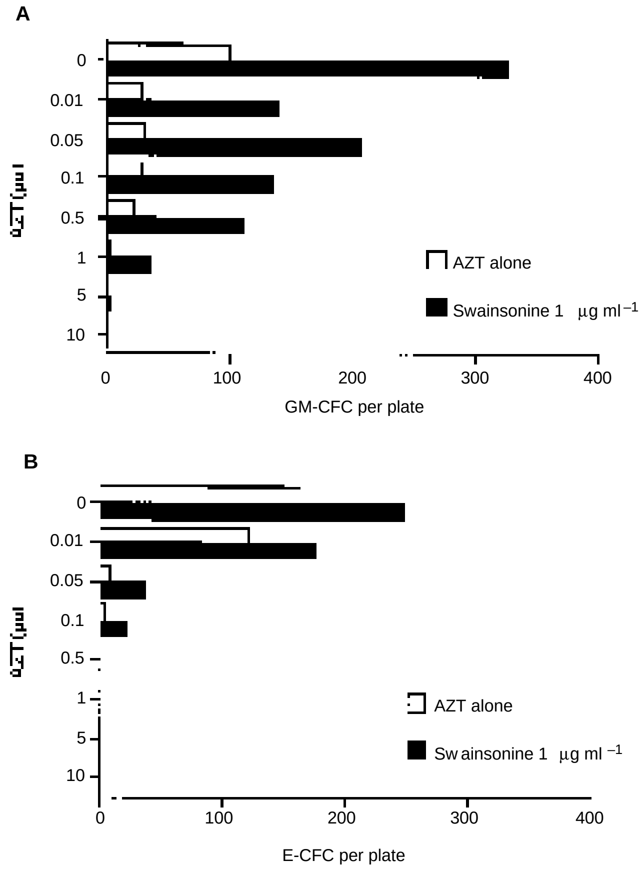

**Figure 4** Effect of swainsonine on murine bone marrow progenitor cell growth in methylcellulose medium in the presence of AZT. AZT alone (□) or with 1 µg ml$^{-1}$ of swainsonine (■) was added to the methylcellulose medium at concentrations indicated on the graphs. After 14 days of incubation, CFC were identified and scored. (**A**) GM-CFC; (**B**) early E-CFC

displayed reduced peripheral red blood cells (RBC), haematocrit, reticulocytes, monocytes and eosinophils (data not shown). The results of an experiment involving 23 days of AZT treatment are shown in Table 2. Swainsonine treatment alone caused a slight, but statistically significant, increase in RBC count and haematocrit, and a slight decrease in reticulocytes. However, swainsonine treatment of mice that received AZT for 23 consecutive days markedly increased the number of white blood cells compared to animals treated with AZT alone. This effect was essentially due to a large increase in the number of lymphocytes in AZT-treated animals that received swainsonine (Table 2).

### Swainsonine effect on the growth of murine bone marrow progenitor cells in culture

To determine whether swainsonine directly affected bone marrow progenitor cells, short-term haematopoietic cultures were used. The addition of swainsonine to the growth medium significantly increased the number of early murine E-CFC (230% of control), with the optimum stimulation at a concentration of 3 µg ml$^{-1}$ (Figure 3). Similarly, swainsonine increased the number of GM-CFC with a maximum increase (210%) at concentrations between 0.03 and 10 µg ml$^{-1}$.

To examine the ability of swainsonine to alter the toxicity of AZT toward bone marrow cells, both agents were added to the culture medium. The results, shown in Figure 4, indicate that AZT decreased the number of both erythroid and myeloid colonies in methylcellulose cultures. For example, 0.5 µM AZT inhibited growth of GM-CFC by about 80%. Erythroid colonies were more sensitive to AZT toxicity, as 0.5 µM AZT completely inhibited E-CFC growth. However, swainsonine protected both myeloid and erythroid progenitor cells from the toxicity of AZT. For example, addition of swainsonine (1 µg ml$^{-1}$) to the medium containing 0.5 µM AZT restored the number of murine GM-CFC to a level similar to the control (Figure 4A).

### Swainsonine effects on human bone marrow progenitor cells treated with AZT in vitro

We next examined the effects of swainsonine on human bone marrow progenitor cells in vitro. In the presence of AZT, swainsonine induced a 20–30% increase in the number of human GM-CFC over that observed with AZT alone in the culture medium (Table 3). This effect was observed for two different concentrations of swainsonine and in bone marrow cultures from four different donors. Further analysis of these results using the isobologram method (Elion et al, 1954) clearly showed that swainsonine antagonized the toxicity of AZT on human bone marrow cells in vitro in the two bone marrow samples which yielded data amenable to this analysis (Figure 5). The protection of human bone marrow progenitor cells was similar to that observed with murine bone marrow cells. These results demonstrated that swainsonine reduced the toxicity of a known myelosuppressive drug on human myeloid progenitor cells.

### Swainsonine effects on haematopoietic cell lines in vitro

Any clinical utility of swainsonine will depend in part on its ability to stimulate haematopoietic cells without harmful side-effects. One potential side-effect, based on the observed stimulation of

 © Cancer Research Campaign 1999

**Table 3** Effects of swainsonine (Sw) on human bone marrow colonies growing in presence of AZT

| Sw concentrations | Average increase over AZT alone |
|---|---|
| 50 μg ml$^{-1}$ | 19.4% ± 3.9 |
| | *P* < 0.01 |
| 100 μg ml$^{-1}$ | 27.6% ± 5.1 |
| | *P* < 0.02 |

The values are the average of the percentage of the increase of the numbers of GM-CFC colonies due to the addition of Sw in the medium containing 1 μM AZT over the number of colonies from the medium containing AZT alone. The *P*-values were calculated by Student's two-tailed paired *t*-test.

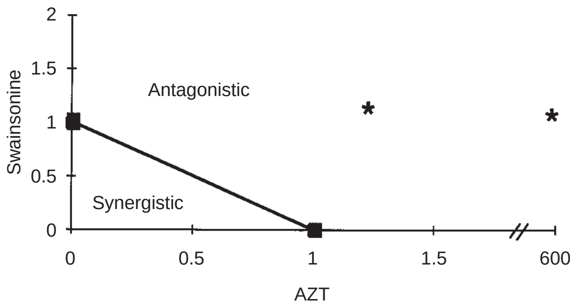

**Figure 5** Isobologram analysis (Elion et al, 1954) of the effects of AZT and swainsonine on human bone marrow colony-forming units. Fractional inhibitory concentrations (FICs) were calculated by dividing the concentration of the drug in the combination by the concentration of the drug alone required to produce the selected inhibition of control growth (here 95%). The two stars represent the relative FICs for AZT in the presence of swainsonine from the two human GM-CFC data sets amenable to this analysis normal haematopoietic cells, is the stimulation of haematopoietic tumour cells. Thus, we measured the effect of swainsonine on several haematopoietic cell lines both in liquid culture and in methylcellulose. Both human (Jurkat and HL-60) and murine (WR19L and WEHI) haematopoietic cell lines were grown in the presence and absence of swainsonine. None of the cell lines showed any change in growth rate or cell number at saturation in liquid culture (data not shown). Neither the WR19L, WEHI nor HL-60 cells showed any change in colony-forming ability in methylcellulose in a medium rich in growth factors. WEHI cells, but not WR19L or HL-60 cells, displayed a two- to three-fold increase in colony forming ability in methylcellulose cultures, but only when the usual growth factors were omitted from the medium. Thus, there does not appear to be any general stimulatory effect of swainsonine for transformed cells of haematopoietic origin.

### DISCUSSION

In this report, we have shown that swainsonine protects both murine and human bone marrow against the toxicity of drugs used in the treatment of cancer and AIDS. These effects were shown in the survival of tumour-bearing animals and haematopoietic cell colony-forming ability following treatment with the clinically-relevant drugs, cyclophosphamide and AZT. Significantly, swainsonine did not block the tumour-killing capacity of cyclophosphamide as monitored by in vitro plating efficiency and inhibition of growth of subcutaneous xenografts of B16-F1 murine melanoma. Swainsonine also antagonized the toxicity of AZT on human bone marrow progenitor cells under conditions that did not stimulate growth of murine or human haematopoietic tumour cells in vitro. These studies represent the first demonstration that swainsonine can be used in combination with a cytotoxic anticancer agent without diminishing the anti-tumour capacity of the drug.

Interestingly, swainsonine did not stimulate the number of murine bone marrow cells in vivo when administered alone. Rather, swainsonine appeared to improve the recovery of bone marrow after haematological injuries (Oredipe et al, 1991). This may be an important aspect of swainsonine's activity, in that highly stimulatory growth factors may actually sensitize bone marrow progenitor cells to genotoxic damage by anti-tumor therapies, which could ultimately lead to increased rates of leukaemia.

The mechanism by which swainsonine enhances survival is not entirely clear. The fact that both melanoma cells in vitro and as a tumour mass in vivo are sensitive to cyclophosphamide in the presence of swainsonine suggests that swainsonine does not activate a general cellular detoxification pathway or block drug transport into tumour cells. The one clear biochemical effect of swainsonine (Elbein et al, 1981; Tulsiani et al, 1982; Tulsiani and Touster, 1983; Olden et al, 1991) is the inhibition of α-mannosidase activity in lysosomes and Golgi apparatus. The resulting changes in protein glycosylation might lead to altered activity of cell-surface receptors. This idea is consistent with the activity of swainsonine observed in in vitro assays. The assay conditions used for growth of the haematopoietic progenitors included the addition of cytokines or a high percentage of bovine fetal serum that is rich in growth factors. However, it has not yet been shown that swainsonine-altered glycosylation in haematopoietic progenitor cells changes the cell's response to cytokines. Further research on the effects of glycosylation inhibitors on progenitor cells is clearly needed.

In vitro swainsonine stimulated both erythroid and myeloid murine progenitor cells. The absence of any strict lineage specificity indicated that swainsonine affects multiple cell types and, therefore, should not lead to the risk of lineage diversion observed with treatments of certain highly specific cytokines (Miles, 1992). This mild stimulatory effect of swainsonine compensates for the toxicity of AZT toward murine bone marrow progenitor cells in vitro. At a concentration of AZT that yields an 80% reduction in GM-CFC, swainsonine restored the number of colonies to a level similar to that observed in the control cultures.

Although the weight adjusted dose of AZT given to mice in these experiments is higher than that used in typical therapy for AIDS patients (1500 mg per patient daily), the dose is only about 50% higher than AZT levels given to humans in high-dose regimens, using an equivalent surface area dose (Zaretsky, 1995). Our results suggest that high doses of AZT may be clinically feasible, given the ability of swainsonine to prevent some aspects of the myelosuppression associated with AZT treatment.

Swainsonine-treated murine haematopoietic cells did not become completely insensitive to AZT toxicity, suggesting that swainsonine does not induce resistance to toxicity of AZT as does interleukin-1 (Abraham et al, 1993). In this sense, swainsonine appears more similar to Epo or GM-CSF in that these cytokines restore the cellularity of AZT-treated bone marrow without actually blocking cytotoxicity (Miles, 1992). The stimulation by

© Cancer Research Campaign 1999

swainsonine of erythroid colonies in vitro correlated with the in vivo observation on circulating red blood cells. Mice treated for 23 days with swainsonine showed a small but significant increase in the number of RBC. However, this stimulation did not protect RBCs from AZT toxicity. In contrast, swainsonine stimulated and protected against AZT toxicity toward erythroid and myeloid progenitor cells in vitro and led to an increase in lymphocytes in the presence of AZT in vivo. As previously reported, murine peripheral blood cells and their progenitors showed a similar sensitivity to AZT (Cronkite and Bullis, 1990; Thompson et al, 1991). AZT treatment of mice for 23 days led to an almost 50% decrease in white blood cell count compared to control animals. When animals received swainsonine simultaneously with AZT, the number of white blood cells was similar to the untreated control. This protective effect was primarily due to the increased number of lymphocytes in peripheral blood. A better understanding of the mechanism by which swainsonine stimulates haematopoietic cells may allow us to identify swainsonine-sensitive cell populations.

The stimulation of normal progenitor cells by swainsonine led to concerns about the potential effect of swainsonine on haematopoietic tumour cells. Our results show that only one of the four haematopoietic tumour cell lines examined was stimulated by swainsonine and, then, only under specific cell culture conditions (in methylcellulose in the absence of added cytokines). From a clinical perspective, these results suggest that swainsonine's activity on haematopoietic cells need not exclude its use in patients with haematopoietic tumours.

This study provides critical information for the potential use of swainsonine in the management of human malignancy. For swainsonine to be used in patients, it is desirable that swainsonine itself be minimally toxic at effective doses. The toxicity of swainsonine in patients with advanced malignancies has been recently examined (Goss et al, 1994). Patients received a continuous i.v. infusion over 5 days with dose levels from 50 to 550 $\mu$g kg$^{-1}$ day$^{-1}$, levels much higher than those used in our in vivo murine study based on an equivalent surface area dose. Swainsonine caused significant clinical toxicity only at the highest dose levels and only in patients with abnormal pre-treatment liver function. These results suggest that swainsonine may not be toxic at doses that yield significant protection from high dose chemotherapy. Our data presented here, combined with the clinical studies, suggest that swainsonine may be a useful adjuvant in treatment protocols that use either high-dose chemotherapy or multi-dose treatments in which myelosuppression is a major concern.

### ACKNOWLEDGEMENTS

We thank Ms Sandra Ward of NIEHS for performing the haematological analysis on the murine samples. We thank Dr Joe Haseman of NIEHS for help with statistical analysis. We thank Dr John Penta and Dr Donald Lombardi, both of NIEHS, for a critical reading of the manuscript.

### REFERENCES

Abraham NG, Chertkov JL, Staudinger R, Jiang S, Lutton JD, Argani I, Levere RD and Kappas A (1993) Long-term bone marrow stromal and hemopoietic toxicity to AZT: protective role of heme and IL-1. *Exp Hematol* **21**: 263–268

Aidoudi S, Guigon M, Lebeurier I, Caen JP and Han ZC (1996) In vivo effect of platelet factor 4 (PF4) and tetrapeptide AcSDKP on haemopoiesis of mice treated with 5-fluorouracil. *Br J Haemotol* **94**: 443–448

Aviles A, Alatriste S, Talavera A, Delgado S and Rosas A (1995) Alternating combination chemotherapy and interferon improves survival in poor prognosis multiple myeloma. *Clin Oncol R Coll Radiol* **7**: 97–101

Calenda V, Sebahoun G and Chermann JC (1992*a*) Modulation of normal human erythropoietic progenitor cells in long-term liquid cultures after HIV-1 infection. *AIDS Res Hum Retroviruses* **8**: 61–67

Calenda V, Tamalet C and Chermann JC (1992b) Transient stimulation of granulopoiesis and drastic inhibition of erythropoiesis in HIV-2-infected long-term liquid bone marrow cultures. *J Acquired Immune Def Syndr* **5**: 1148–1157

Calenda V, Silvy F and Chermann JC (1993) Effects of thymus humoral factor gamma-2 on lymphohaematopoietic progenitor cells: an in vitro study. *Res Immunol* **144**: 395–406

Cronkite EP and Bullis J (1990) In vivo toxicity of 3′-azido-3′-deoxythymidine (AZT) on CBA/Ca mice. *Int J Cell Cloning* **8**: 332–345

Davey RT, Davey VJ, Metcalf JA, Zurlo JJ, Kovacs JA, Falloon J, Polis MA, Zunich KM, Masur H and Lane HC (1991) A phase I/II trial of zidovudine, interferon-α, and granulocyte–macrophage colony-stimulating factor in the treatment of human immunodeficiency virus type 1 infection. *J Infect Dis* **164**: 43–52

Donadieu J, Boutard P, Bernatowska E, Tchernia G, Couillaud G, Philippe N and Le Gall E (1997) A European phase II study of recombinant human granulocyte colony-stimulated factor (lenograstim) in the treatment of severe chronic neutropenia in children. *Eur J Ped* **156**: 693–700

Dorr RT and Fritz WL (1980) *Cancer Chemotherapy Handbook*. Elsevier North Holland, Inc: New York

Elbein AD, Solf R, Dorling P and Vosbeck K (1981) Swainsonine: an inhibitor of glycoprotein processing. *Proc Natl Acad Sci USA* **78**: 285–295

Elion GB, Singer S and Hitchings GH (1954) Antagonists of nucleic acid derivatives. VIII. Synergism in combinations of biochemically related antimetabolities. *J Biol Chem* **208**: 477–488

Fischl M, Galpin JE, Levine JD, Groopman JE, Henry DH, Kennedy P, Miles S, Robbins W, Starrett B, Zalusky R, Abels R, Tsai HC and Rudnick SA (1990) Recombinant human erythropoietin for patients with AIDS treated with zidovudine. *N Engl J Med* **322**: 1488–1493

Gisselbrecht C, Haioun C, Lepage E, Bastion Y, Tilly H, Bosly A, Dupriez B, Marit G, Herbrecht R, Deconinck E, Marolleau J-P, Yver A, Dabouz-Harrouche F, Coiffier B and Reyes F (1997) Placebo-controlled phase III study of lenograstim (glycosylated recombinant human granulocyte colony-stimulating factor) in aggressive non-Hodgkin's lymphoma: Factors influencing chemotherapy administration. *Leuk Lymph* **25**: 289–300

Goss PE, Baptiste J, Fernandes B, Baker M and Dennis JW (1994) A study of swainsonine in patients with advanced malignancies. *Cancer Res* **54**: 1450–1457

Hoagland CH (1992) Hematologic complications of cancer chemotherapy. In *The Chemotherapy Source Book*, Perry MC (ed), pp. 498–507. Williams & Wilkins: Baltimore

Humphries MJ, Matsumoto K, White SL and Olden K (1986) Oligosaccharide modification by swainsonine treatment inhibits pulmonary colonization of B16-F10 murine melanoma cells. *Proc Natl Acad Sci USA* **83**: 1752–1756

Hunter MG, Bawden L, Brotherton D, Graig S, Cribbes S, Czaplewski LG, Dexter TM, Drummond AH, Gearing AH, Heyworth CM, Lord BI, McCourt M, Varley PG, Wood LM, Edwards RM and Lewis PJ (1995) BB-10010: an active variant of human macrophage inflammatory protein-1α with improved pharmaceutical properties. *Blood* **86**: 4400–4408

Laporte JP, Fouillard L and Dovay L (1991) GM-CSF instead of autologous bone marrow transplantation after BEAM regimen. *Lancet* **338**: 601–602

Lord BI, Dexter TM, Clements JM, Humter MA and Gearing AJH (1992) Macrophage-inflammatory protein protects multipotent hematopoietic cells from the cytotoxic effects of hydroxyurea in vivo. *Blood* **79**: 2605–2609

McLeod GX and Hammer SM (1992) Zidovudine: five years later. *Ann Int Med* **117**: 487–501

Miles SA (1992) Hematopoietic growth factors as adjuncts to antiretroviral therapy. *AIDS Res Hum Retroviruses* **8**: 1073–1080

Olden K, Breton P, Grzegorzewski K, Yasuda Y, Gause BL, Oredipe OA, Newton SA and White SL (1991) The potential importance of swainsonine in therapy for cancers and immunology. *Pharmacol Ther* **50**: 285–290

Oredipe OA, White SL, Grzegorzewski K, Gause BL, Cha JK, Miles VA and Olden K (1991) Protective effects of swainsonine on murine survival and bone marrow proliferation during cytotoxic chemotherapy. *J Natl Cancer Inst* **83**: 1149–1156

Perry MC (1992) *The Chemotherapy Source Book*. Williams & Wilkins: Baltimore

Pratt WB, Ruddon RW, Ensminger WD and Maybaum J (1994) *The Anticancer Drugs*. Oxford University Press: New York

Richman DD, Fischl MA, Grieco MH, Gottlieb MS, Volberding PA, Laskin OL, Leedom JM, Groopman JE, Mildvan D, Hirsch MS, Jackson GG, Durack DT, Phil D and Nusinoff-Lehrman S (1987) The toxicity of azidothymidine (AZT) in the treatment of patients with AIDS-related complex. *N Engl J Med* **317**: 192–197

© Cancer Research Campaign 1999

Shaunak S and Bartlett JA (1989) Zidovudine-induced neutropenia: are we too cautious? *Lancet* **I**: 91–92

Thompson MB, Dunnick JK, Sutphin ME, Giles HD, Irwin RD and Prejean JD (1991) Hematologic toxicity of AZT and ddC administered as single agents and in combination to rats and mice. *Fund App Toxicol* **17**: 159–176

Tulsiani DRP, Harris T and Touster O (1982) Swainsonine inhibits the biosynthesis of complex glycoproteins by inhibition of golgi mannosidase II. *J Biol Chem* **257**: 7936–7939

Tulsiani DRP and Touster O (1983) Swainsonine causes the production of hybrid glycoproteins by human skin fibroblasts and rat liver Golgi preparations. *J Biol Chem* **258**: 7578–7585

Vose JM and Armitage JO (1992) Bone marrow transplantation. In *The Chemotherapy Source Book*, Perry MC (ed), pp. 498–507. Williams & Wilkins: Baltimore

Walker R, Parker R, Kovacs JA, Masur H, Gralnick H, Lane HC and Fauci AS (1987) 3′-Azido-3′-deoxythymidine causes red cell aplasia and hypoplasia in AIDS patients with Kaposi's sarcoma. *Clin Res* **35**: 435A

Watanabe T, Kelsey LS, Yan Y, Brown GS, Jackson JD, Ewel C and Talmadge JE (1996) In vivo haemoprotective activity of tetrapeptide AcSDKP combined with granulocyte-colony stimulating factor following sublethal irradiation. *Br J Haematol* **94**: 619–627

Yarchoan R, Mitsuya H, Myers CE and Broder S (1989) Clinical pharmacology of 3′-azido-2′,3′-dideoxythymidine (zidovudine) and related dideoxynucleosides. *N Engl J Med* **321**: 726–773

Zaretsky MD (1995) AZT toxicity and AIDS prophylaxis: Is AZT beneficial for HIV+ asymptomatic persons with 500 or more T4 cells per cubic millimeter? *Genetica* **95**: 91–101

*© Cancer Research Campaign 1999*
